# Editorial Note: Multi-objective optimization of dual resource integrated scheduling problem of production equipment and RGVs considering conflict-free routing

**DOI:** 10.1371/journal.pone.0353811

**Published:** 2026-07-15

**Authors:** The PLOS One Editors

The *PLOS One* Editors issue this Editorial Note because this article [1] was identified as one of a series of submissions for which we have concerns related to the peer review process. Readers are encouraged to approach the article [1] with careful consideration. In following up on this matter, PLOS did not evaluate the article’s integrity or scientific validity.

The Editors do not have concerns regarding the authors’ conduct during the peer review process.
